# What makes a rhythm complex? The influence of musical training and accent type on beat perception

**DOI:** 10.1371/journal.pone.0190322

**Published:** 2018-01-10

**Authors:** Fleur L. Bouwer, J. Ashley Burgoyne, Daan Odijk, Henkjan Honing, Jessica A. Grahn

**Affiliations:** 1 Institute for Logic, Language and Computation, University of Amsterdam, Amsterdam, The Netherlands; 2 Amsterdam Brain and Cognition, University of Amsterdam, Amsterdam, The Netherlands; 3 Informatics Institute, University of Amsterdam, Amsterdam, The Netherlands; 4 Brain and Mind Institute, Department of Psychology, University of Western Ontario, London (ON), Canada; Max Planck Institute for Human Cognitive and Brain Sciences, GERMANY

## Abstract

Perception of a regular beat in music is inferred from different types of accents. For example, increases in loudness cause intensity accents, and the grouping of time intervals in a rhythm creates temporal accents. Accents are expected to occur on the beat: when accents are “missing” on the beat, the beat is more difficult to find. However, it is unclear whether accents occurring *off* the beat alter beat perception similarly to missing accents *on* the beat. Moreover, no one has examined whether intensity accents influence beat perception more or less strongly than temporal accents, nor how musical expertise affects sensitivity to each type of accent. In two experiments, we obtained ratings of difficulty in finding the beat in rhythms with either temporal or intensity accents, and which varied in the number of accents on the beat as well as the number of accents off the beat. In both experiments, the occurrence of accents on the beat facilitated beat detection more in musical experts than in musical novices. In addition, the number of accents on the beat affected beat finding more in rhythms with temporal accents than in rhythms with intensity accents. The effect of accents off the beat was much weaker than the effect of accents on the beat and appeared to depend on musical expertise, as well as on the number of accents on the beat: when many accents on the beat are missing, beat perception is quite difficult, and adding accents off the beat may not reduce beat perception further. Overall, the different types of accents were processed qualitatively differently, depending on musical expertise. Therefore, these findings indicate the importance of designing ecologically valid stimuli when testing beat perception in musical novices, who may need different types of accent information than musical experts to be able to find a beat. Furthermore, our findings stress the importance of carefully designing rhythms for social and clinical applications of beat perception, as not all listeners treat all rhythms alike.

## Introduction

In musical rhythm, we often perceive a regular beat. The beat is what we tap our feet to, and the perception of a beat in music makes some musical events sound more prominent than others. To perceive a beat in a rhythm, we rely on various types of *accents* [1,2]. An accent is an acoustic event that is more salient than its surrounding context. Salience can be caused by differences in pitch, intensity or timbre [3], in which case an accent is present in the physical properties of the sound, but it can also arise from variation in the grouping structure of a rhythm [4]. When accents occur at regularly spaced points in time, a listener can perceive a beat in a rhythm [2], and the beat generally coincides with accented events [1]. Initially, when hearing a rhythm, a listener needs to detect its regularity to find the beat [5]. Once a beat has been inferred from a rhythm, its perception remains stable [6], and thereafter the beat can coincide with silence, or an accent can even occur off the beat, as in a syncopation [2], without beat perception being too disrupted. The relationship between the structure of accents in music and the perceived beat is thus flexible, and as such, the perception of a beat is regarded as a psychological construct [6–9]. A beat is often embedded in a hierarchical organization with several nested levels of perceived regularity, the metrical structure. Within the metrical structure, the beat is the most salient level of regularity. The faster regularity at a hierarchically lower level than the beat is termed a *subdivision* of the beat. In turn, the beat can be a subdivision of a slower, higher-order regularity of more and less salient beats, which is sometimes referred to as *meter*.

The flexible relationship between the presence of accents in a rhythm and the perceived beat has been described in the context of general predictive processing in the brain [10,11]. At any point while listening to a rhythm, a listener will compare a top-down internal model of the metrical structure with the incoming bottom-up information. A mismatch between the bottom-up input and the top-down model leads to updating of the model, to arrive at better predictions of the upcoming rhythm [10,11]. Initially, when trying to find a beat in a rhythm, a listener will rely on an internal model based on previous experience. In Western culture, listeners are thought to initially expect a rhythm in duple meter, as this is the most frequently used metrical structure [10,11]. Listeners will predict that future accented events are likely to occur on the beat [5].

A variety of stimuli have been used to study beat perception, ranging from isochronous sequences [12–16] to rhythms with varying inter-onset intervals but identical sounds [17–19], rhythms with varying acoustic properties but with identical inter-onset intervals [3,20–24], and real music [25–27]. Stimuli may contain various types of accents that indicate the beat to a listener. Accents can be created by varying the grouping structure or acoustic features of a rhythm, and the structure of such accents has been shown to contribute to beat perception (cf. [28], intensity differences; [3], duration and pitch; [29], pitch; [30], grouping structure). In real music, multiple types of accents determine the salience of rhythmic events [31].

Although it is well established that different types of accents contribute to beat perception, it is unclear whether these different accents contribute to beat perception in differing ways (i.e., are some accents more influential than others, and if so, which?). It is also unknown whether mismatches between the accent structure and the perceived beat are perceived similarly on the beat versus off the beat (i.e., does an unexpected missing accent on the beat have the same effect on the perception of the beat as an unexpected accent off the beat?). In the current study, we address these issues by examining the contributions to beat perception of two types of accents: *temporal accents* and *intensity accents*. In addition, we explore whether musical expertise affects how sensitive a listener is to the structure of accents in a rhythm. Higher sensitivity to the structure of the accents and their relation to the metrical structure is expected to lead to both easier finding of a beat and more sensitivity to possible mismatches between the accent structure and a perceived beat. Thus, if musical training leads to higher sensitivity to the accent structure, we would expect musical experts to differentiate more than musical novices between rhythms that contain a clear beat and rhythms in which the accents violate the metrical structure.

Temporal accents arise from the grouping structure of the time intervals between events (e.g., note onsets) that make up a rhythm. Rhythmic events are perceived as accented when they are isolated in time, the second of a group of two events, or the first or last of a group of three or more events [4]. Temporal accents are thus not caused by physical properties of the sounds in a rhythm, but rather by the way the sounds are grouped together. The relation between the perceived beat and the structure of temporal accents has been described by Povel and Essens [30] with a complexity score, which is a weighted sum of all beats that do not contain an event and all beats that contain an event but are unaccented. The complexity score is thus a measure of counterevidence against a possible perceived beat and indicates how well a given rhythm fits with the perception of a certain beat. Many studies examining beat perception have used rhythms with temporal accents (hereafter: *temporal rhythms*), designed after the Povel and Essens model [30], and the relationship between the number of unaccented beats and difficulty in perceiving the beat is well established [17–19,32].

Contrary to *counterevidence on the beat* (i.e., silences or unaccented events on the beat), *counterevidence off the beat* (i.e., accents occurring between beats) is not taken into account by Povel and Essens [30]. This is in line with the dynamic attending theory (DAT; [33]). DAT proposes that fluctuations in attentional energy can entrain their phase and period to an external rhythm. Peaks in attentional energy then coincide with the beat, leading to more sensitivity to sensory input that coincides with the beat than to input that falls between beats. However, several studies have shown that unexpected intensity increases, which are accents that are physically present in the sound of a rhythm, are more salient off the beat than on the beat [34–37]. Accents off the beat may be more salient than on the beat as they disrupt the regularity of the perceived beat. Similar to missing accents on the beat, accents off the beat can be interpreted as counterevidence against a perceived beat. DAT suggests that we are more sensitive to information on the beat than off the beat. However, the salience of intensity accents off the beat raises the question whether counterevidence off the beat may also contribute to beat perception, and whether temporal accents off the beat, caused by the grouping structure of a rhythm, are as disruptive as intensity accents.

Unlike the relationship between missing temporal accents on the beat and the beat that is perceived, which has been described by the Povel and Essens model [30], the relationship between the structure of intensity accents and the beat that is perceived has not been formalized. Despite this lack of formal characterization, many studies have used rhythms with intensity accents (hereafter: *intensity rhythms*) to induce a beat [20,21,28,37–39], and models and theories of beat perception stress the importance of intensity accents [2,40,41].

In one study, responses to temporal and intensity rhythms were compared directly [42]. Beat perception was examined in musicians and non-musicians in response to both types of rhythms using behavioral methods and fMRI. The beat was rated to be more salient in intensity rhythms than in temporal rhythms. However, temporal rhythms elicited more activity than intensity rhythms in the supplementary motor area and the basal ganglia, two brain areas associated with beat perception [7,9,17]. Thus, listeners appeared to process temporal and intensity accents differently. In addition, musicians showed greater connectivity between premotor areas and auditory cortex than non-musicians while listening to temporal rhythms that contained a beat, but not while listening to intensity rhythms that contained a beat. Thus, in addition to general processing differences between temporal and intensity rhythms, musical training may selectively increase sensitivity to the structure of accents indicating the beat in temporal, but not intensity rhythms.

Note that in addition to temporal and intensity accents, which are driven by stimulus features, we often perceive some events as more salient than others, even when no physical accents are present in a rhythm. For example, in an isochronous sequence of tones, listeners may spontaneously hear a binary beat (e.g., ‘tick-tock-tick-tock’), with alternating accented and unaccented tones [15,43], even though the tones are acoustically identical. Such accents, known as metrical accents, are likely caused by our internal representation of the beat: once we establish an internal sense of the metrical structure, we interpret incoming sounds in the context of that structure and perceive some sounds as more salient than others, consistent with the internal representation. Thus, while intensity and temporal accents are used to infer a beat from rhythm, and cause us to update our internal representation of the metrical structure, metrical accents are a reflection of the perceived beat, not its cause (cf. [11,44]). From this it follows that counterevidence against an already perceived beat can also be regarded as a mismatch between the structure of accents present in the rhythm (i.e., temporal and intensity accents) and the structure of accents present in the mind of the listener (i.e., metrical accents).

Although beat perception develops spontaneously in humans [9], individuals vary widely in their ability to extract a beat from musical rhythm [45,46]. Some of this variability may result from musical training, which enhances beat perception abilities [23,36,47]. Based on the fMRI findings described above, these musical training enhancements may depend on the type of accents present in the rhythm. Enhanced beat perception abilities in musical experts may stem from more exposure to rhythm, which may lead to stronger prior expectations about metrical structure [10,11]. Additionally, it has been shown that musically trained subjects use a different strategy when listening to rhythm than untrained subjects. Whereas untrained subjects tend to focus on and tap to lower (faster) levels in the metrical hierarchy, musically trained subjects more often focus on and tap to higher order regularities in the hierarchy [48,49].

In the current study, we aimed to examine the contributions of different kinds of accents to beat perception in musical experts and musical novices. First, we compared the influence of temporal accents and intensity accents on beat perception. Second, we examined the effects of the occurrence of accents both on the beat and off the beat. Finally, we looked at the influence of musical training. As in previous studies [17–19], we constructed temporal rhythms with varying metrical complexity based on Povel and Essens [30]. However, contrary to previous studies, we not only manipulated how many accents occurred on the beat, but we also varied how many accents occurred off the beat.

We constructed intensity rhythms that mirrored the temporal rhythms in terms of the number of accents on and off the beat. To create identical patterns of accents in the intensity condition as in the temporal condition, the intensity condition was composed of evenly spaced tones at the lowest (fastest) level of the metrical hierarchy, with intensity accents placed on certain tones, mirroring the pattern of temporal accents in the temporal condition. Thus, in the intensity condition, only differences in intensity marked accented events, not differences in temporal grouping of tones.

Note that we refer to tones as being “on the beat” and “off the beat” in relation to the duple beat we expected participants to find in the rhythms. In general, listeners are biased to hear a duple beat, based on exposure to Western music [10,11]. To further reinforce a duple meter, we used rhythms with 16 events (cf. [30]). While we are aware that for some rhythms, participants may not be able to find a beat, to describe the structure of the accents, we refer to accents as either on the beat or off the beat in relation to a duple metrical structure.

In Experiment 1, using a web-based setup, we obtained ratings of beat perception difficulty for intensity and temporal rhythms (which contained varying numbers of accents on and off the beat) from participants with different levels of musical expertise. While previous studies have used the accuracy of tapping to a beat as a measure of how difficult is it to perceive a beat in a rhythm, using ratings of subjective experience provides two advantages. First, tapping to a beat and rating a rhythm for beat presence may in fact be related to the strength of a beat in different ways. Tapping relies on predicting the beat, as taps are initiated before an onset occurs, and syncopation can only affect taps that occur after the syncopation, not before. Rating rhythms for beat presence allows for the effect of the syncopation to be taken account into the final rating, as it occurs at the end of the rhythm. Indeed, when both response measures were directly compared in a study looking at the perception of syncopated rhythms, ratings were found to be more sensitive to violations of the metrical structure than tapping [50]. Second, and perhaps more important, moving to a beat can alter the perception of the strength of that same beat, increasing the strength of the beat percept relative to when the rhythm is perceived without tapping along [51]. As we are primarily interested in the perception of a beat, we therefore chose to use a measure for perceived beat strength that did not require movement (see [52] for a similar approach). In Experiment 2, we validated the results from Experiment 1 using a second, more constrained set of rhythms.

We expected that a larger mismatch between the structure of accents and the perceived beat (e.g., increased counterevidence, both on the beat and off the beat) would increase the difficulty of perceiving a beat, in both temporally accented and intensity accented rhythms. We also expected musical training to selectively enhance the sensitivity to the structure of the accents in rhythms with temporal, but not intensity accents, based on previous work [42]. In the intensity rhythms, all subdivisions (the lowest level of the metrical hierarchy) are marked by a tone, and musical novices may use a listening strategy aimed at the regularity at this level [49]. The lack of marked subdivisions in the temporal rhythms may make beat finding more difficult in these rhythms for musical novices. Finally, we expected intensity accents to be more salient than temporal accents, and thus to be more perturbing of beat perception than temporal accents when used as counterevidence off the beat.

## Experiment 1

### Methods

#### Participants

The data reported here was retrieved from the online application on February 6, 2015. At that time, a total of 91 people had viewed the start page of the online application for Experiment 1, of whom 78 people had provided consent, 72 had provided their age and years of musical training, 56 had finished reading the instructions, and 54 had listened to the examples. Finally, 48 participants had proceeded to rate one or more rhythms (for more details, see the Procedure section). To improve reliability, 16 participants who rated fewer than 60 rhythms were considered dropouts and were excluded. The dropout rate was thus 33 percent, which is comparable to previous online music cognition experiments (cf. [53]). The remaining 32 participants were on average 33.3 years old (range 18–66 years, SD = 14.5) and reported on average 11.1 years of musical training (range 0–25 years, SD = 8.3).

Before starting the experiment, participants were shown a screen with information about the study. At the bottom of the screen, the following text was shown: “You have read the Letter of Information and have had the nature of the study explained to you. By clicking the button below, you agree to participate.” To provide informed consent and start the experiment, participants clicked a button with the text “I agree, continue to experiment”. The study and this procedure to obtain consent from participants in this online study were approved by the Ethics Committee of the Faculty of Humanities of the University of Amsterdam and the Non-Medical Research Ethics Board of the University of Western Ontario.

#### Stimuli

**Fig 1:**
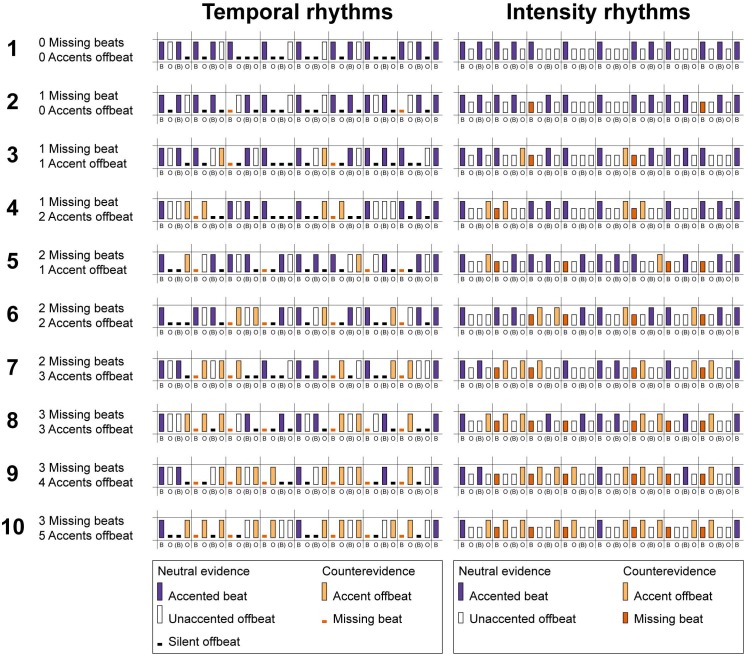
Examples of rhythms for each condition. Each rhythm as used in the experiment is constructed from two of the original 16 grid-point rhythms, followed by a final tone, for a total of 33 grid points. The spacing between the two halves of the rhythm and before the final tone is for viewing purposes only. In the concatenation of the rhythms, the isochronicity of the grid-points was preserved. Note that the number of missing beats and number of accents off the beat refer to counterevidence in a rhythm of 16 grid-points. Sound examples for each condition are available as Supporting Information. S1–S10 Sounds are examples of temporal rhythms from conditions 1–10 respectively, and S11–S20 Sounds are examples of intensity rhythms from conditions 1–10 respectively. B = beat (positions 1, 5, 9, and 13); O = off the beat (positions 2, 4, 6, 8, 10, 12, 14, and 16); (B) = ambiguous (positions 3, 7, 11, and 15; off the beat when subdivided into four beats of four sixteenth notes; on the beat when subdivided into eight beats of two eighth notes).

We generated all possible rhythms of 9 tones and 7 silences aligned to a grid of 16 positions, with the grid positions representing four beats subdivided into four sixteenth tones (see Fig 1). Note that in the experiment, we always presented participants with two concatenated rhythms consisting of two rhythms, each with 16 positions, as an initial pilot showed that the rhythms with only 16 grid positions were too short for people to make judgments about the difficulty of perceiving a beat. Here, we will first explain how we constructed the rhythms with 16 positions, before explaining how the final selection of the concatenated rhythms was made.

By using 16 grid points, which can be divided into groups of two or four, but not into groups of three, we reinforced the perception of a binary metrical structure [30]. We selected a binary metrical structure because the beat is easier to perceive in a binary than in a ternary metrical structure [54], and because we expected that listeners would have an a priori expectation for duple metrical structures. Positions 1, 5, 9 and 13 were considered to be on the beat. We assigned temporal accents to events based on [30], with isolated events, the second of two consecutive events and the first and last of three or more consecutive events considered accented. Temporal rhythms were subsequently selected based on five constraints. First, only patterns that started with an event were considered. Second, in order to avoid unevenly distributed patterns, we allowed a maximum of five consecutive events and a maximum of three consecutive silences. Third, in order to avoid too much repetition in the rhythms, we only included rhythms in which the four sixteenth notes that made up each of the four beats (notes 1–4, 5–8, 9–12, and 13–16 for the four respective beats) contained a different configuration of events, Thus, rhythms in which multiple beats consisted of the same pattern (for example one eighth note and two sixteenth notes, repeated four times) were not included. Fourth, only patterns with six accented events were used. Finally, as was done previously [18], temporal rhythms with unaccented beats were excluded, allowing silence to be the only type of counterevidence on the beat.

For each rhythm, the number of accents on and off the beat were counted. As the first position always contained an event, the number of accents on the beat (positions 1, 5, 9, and 13) varied between 1 and 4. As we excluded unaccented beats, any beat that was not accented was automatically silent. Thus, the number of beats missing varied from 0 to 3. We characterized the rhythms by the number of beats missing, or counterevidence on the beat, as this is comparable to the original model by Povel and Essens [30]. However, note that in the current experiment, the results for beats missing can be interpreted equally well as reflecting the influence of the number of accents present on the beat (positive evidence [55]).

Although we designed the rhythms to be perceived as four beats subdivided into four sixteenth tones, it is possible to hear a rhythm consisting of 16 grid-points as eight beats subdivided into two eighth tones. We chose a tempo that was optimal for hearing the rhythms as containing four beats, or one beat every four grid points (see below), but we did not want to exclude the possibility that listeners would perceive the patterns as containing eight beats, or one beat every two grid points. Positions 3, 7, 11 and 15 could either be off the beat (if four beats were perceived) or on the beat (if eight beats were perceived). We did not want to make assumptions about the metrical level that listeners would hear a beat at and therefore we did not know whether accents in these positions should be regarded as positive or negative evidence. Thus, we did not count evidence in these ambiguous positions (3, 7, 11, and 15). Therefore, the number of accents off the beat was counted as the number of accents in all even-numbered positions.

Intensity rhythms were constructed to be analogous to the temporal rhythms (see Fig 1). Each position on the grid was filled with a tone and intensity accents were introduced on the same positions where temporal accents occurred in the temporal rhythms. Thus, like the temporal rhythms, all intensity rhythms contained six accents. However, unlike the temporal rhythms, in the intensity rhythms a sound occurred on each subdivision of the beat. While the temporal rhythms contained three different event types (accented events, unaccented events and silences), the intensity rhythms only contained two different types (accented and unaccented events). The accented events were always in the same positions for the two types of rhythms, but unaccented events in the intensity rhythms could map onto either unaccented events or silences in the temporal rhythms. Thus, different temporal rhythms could map onto the same intensity rhythm. Therefore, while a total of 670 temporal rhythms adhered to our criteria, only 120 intensity rhythms were possible with the current constraints. Also, within the constraints concerning the total number of accents and events, some combinations of missing beats and accents off the beat were not possible and others were unlikely. For example, when three beats are missing (i.e., position 1 is accented, but positions 5, 9, and 13 are silent), it is impossible to have six accents that do not occur off the beat (e.g., only positions 1, 3, 7, 11, and 15 are not considered off the beat, which is not enough to meet our requirement to have six accents in each rhythm). To be able to test our hypotheses with several different rhythmic patterns per condition, we only included the ten conditions that allowed for six or more different rhythmic patterns (see Table 1).

**Table 1:** Characteristics of the rhythms used in Experiment 1.

Missing beats	Accents off the beat	Possible 16 grid-point rhythms		Number of concatenated 32 grid-point rhythms used in Experiment 1		Description of number of accents off the beat	No
		Temporal	Intensity	Temporal	Intensity		
**0**	0	12	6	24	24	Few	1
**1**	0	36	6	15	15	Few	2
	1	98	18	15	15	Some	3
	2	56	6	15	15	Many	4
**2**	0	11	3	0	0	Not used	
	1	65	12	15	15	Few	5
	2	143	22	15	15	Some	6
	3	111	16	15	15	Many	7
	4	18	3	0	0	Not used	
**3**	2	10	2	0	0	Not used	
	3	37	8	12	12	Few	8
	4	53	12	12	12	Some	9
	5	20	6	10	10	Many	10
**Total number of rhythms**				296			

The leftmost four columns of the table show how many 16 grid-point rhythms were possible with the constraints used for the construction of the rhythms. For some combinations of counterevidence on and off the beat, only a few intensity rhythms could be constructed. To ensure sufficient variety in the stimulus material, conditions with fewer than 6 possible rhythms were not included in the experiment. These conditions are indicated as “Not used”. The right half of the table shows the number of rhythms in each condition that were included in the experiment and the description of the number of accents off the beat we used in the analysis. The numbers in the rightmost column correspond to the numbering for the conditions used in Fig 1. In the Supporting Information, S1–S10 Sounds are temporal rhythms and S11–S20 Sounds are intensity rhythms corresponding to rhythms from conditions with numbering 1–10 in both Fig 1 and the rightmost column of this table.

To make it easier for participants to judge beat presence in the rhythms, we constructed longer rhythms for each condition by concatenating pairs of different semi-randomly selected rhythms with the same number of missing beats and the same number of accents off the beat into rhythms of 32 grid-points. The randomization was optimized to create as much variety as possible in the rhythms. A final tone was appended to each rhythm to provide metrical closure [18]. Fig 1 shows an example rhythm for each condition. Sound examples for rhythms from each condition are available as Supporting Information S1–S20 Sounds. During the experiment, participants were specifically asked to detect a beat in the rhythms. Only one of the ten conditions contained strictly metric rhythms (i.e., without any counterevidence). The inclusion of counterevidence may make it hard to hear a beat, especially for musical novices. To prevent them from getting discouraged during the experiment, we did not include an equal number of rhythms from each condition in the experiment, but rather used a larger number of rhythms from the condition without counterevidence than from each condition with counterevidence. Table 1 shows the total number of rhythms used for each condition. Fig 1 and Table 1 have the same numbering for the ten conditions used, which correspond to Supporting Information S1–S10 Sounds (examples of temporal rhythms) and S11–S20 Sounds (examples of intensity rhythms).

All sounds were woodblock sounds generated in Garageband (Apple Inc.). For the intensity rhythms, the difference between accented and unaccented events was set to 8.5 dB, comparable to the intensity rhythms in Grahn and Rowe [42]. Intensity and temporal rhythms were equated for overall loudness by scaling all sounds in the temporal rhythms to 0.8 dB softer than the accented sounds in the intensity rhythms. The inter-onset interval between grid points was varied across rhythms to prevent carryover of the perceived beat from one trial to the next. A tempo of around 100 beats per minute (inter-beat interval of 600 ms) is the optimal tempo for human adults to perceive a beat at [48,56]. Assuming a subdivision of the rhythms into beats of four sixteenth tones, this would correspond to an inter-onset interval of 150 ms between grid-points. For each rhythm, one of five inter-onset intervals around this rate was used (140, 145, 150, 155 and 160 ms), corresponding to tempi of 107, 103, 100, 97 and 94 beats per minute.

#### Procedure

A web-based application to rate auditory stimuli was created using the Google App Engine (Google Inc.). To foster future research, we have released this application as open-source software at https://github.com/dodijk/annotate. For viewing purposes, the application can be accessed online at http://uvamcg.appspot.com. When accessing the website, participants were presented with four obligatory steps before the experiment started. First, they provided informed consent. Second, they provided their age in years and the number of years of formal musical training they had received in their life. Third, they were presented with a written explanation of the experiment. Finally, they were presented with example rhythms. Participants were asked to perform the experiment in a quiet environment and use a computer rather than a mobile device. They received an explanation of the term “beat” and were given the following instructions: “For each rhythm, we ask you to rate on a scale of 1–10 how hard you think it would be to tap along with the beat in that rhythm. Rate each rhythm by clicking on the stars.” They were presented with examples of both temporal and intensity rhythms with no missing beats and no accents off the beat (e.g., strictly metric rhythms), which contained the caption “This is an example of a rhythm containing a clear beat, which sounds easy to tap along to. We expect people to give this rhythm 1 star.” Examples of temporal and intensity rhythms with three beats missing and several accents off the beat were presented accompanied by the caption “This is an example of a rhythm NOT containing a clear beat, which sounds hard to tap along to. We expect people to give this rhythm 10 stars.” Participants could listen to the examples as often as they liked. After listening to the examples, participants could continue with the experiment.

The interface used for the rating task can be found in S1 Fig. Participants were presented once with each rhythm, at a tempo randomly chosen from the five tempi used. After each rating, the application automatically continued with the next rhythm. Once loaded, each rhythm was preceded by 500 ms of silence to allow participants to focus on the start of the trial. After every 30 rhythms (about 5 minutes), a screen appeared indicating a break. Participants could continue the experiment at their own discretion. If a participant rated all 296 rhythms once, the experiment automatically quit with a screen thanking the participant for their time. The number of rhythms a participant rated therefore varied between 1 (for participants who dropped out immediately) to 296 (for participants who completed the full experiment).

#### Statistical analysis

**Fig 2:**
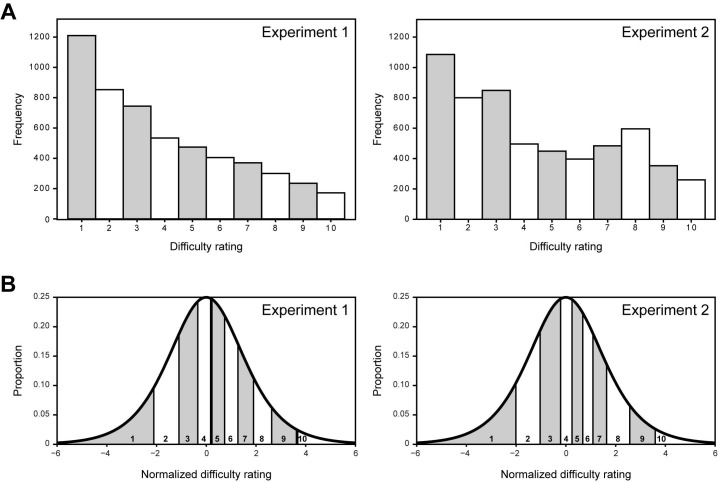
Distribution and normalization of ratings. A) Histograms of ratings from Experiment 1 and 2. B) Normalizations obtained with the ordinal regression for Experiment 1 and 2. The area under the curve for each rating corresponds to the proportion of responses for that rating.

In total, 5578 ratings were made. After excluding participants who rated less than 60 rhythms, 5297 ratings were included in the analysis. The distribution of the ratings is shown in Fig 2A. The distribution is skewed leftwards, indicating a bias for participants to provide low ratings. Further inspection of the data showed that this bias was not caused by the fact that a greater number of strictly metric rhythms were presented than rhythms with some syncopation, but instead that the skewed distribution was present for ratings in each individual condition. Such a distribution is often observed for Likert-items [57]. In general, responses on Likert items can be considered ordinal [58], especially when only one item is used [59]. Because the rating scales used in the experiment were ordinal and not interval, and to correct for the skewedness of the distribution, which prohibits the use of parametric statistical tests, we used a mixed ordinal regression model for our analysis. This technique corrects for potentially unequal distances between the observed rating points on a normalized scale of perceived difficulty. The results of an ordinal regression can be interpreted similarly to results from a normal regression, but the ordinal regression corrects for the data being non-normal. The normalization of the raw ratings obtained through the ordinal regression is depicted in Fig 2B. Note that while the raw ratings ranged from 1 (very easy) to 10 (very hard), for the normalized difficulty ratings negative numbers indicate ratings for rhythms that were judged as easy, while positive numbers indicate ratings for rhythms that were judged as hard.

The normalized ratings served as the dependent variable in the regression model. We used a mixed model, including both fixed and random effects. Four independent variables were included as fixed factors: missing beats, accents off the beat, type, and musical training. *Missing beats* was defined as the number of beats that were silent (temporal rhythms) or unaccented (intensity rhythms) in each 16 grid-point rhythm (see Fig 1). The number of missing beats ranged from 0 to 3 (corresponding to a range of 4 to 1 accents on the beat). The number of *accents off the beat* ranged from 0 to 5. With the constraints that we put on the rhythms, the absolute number of accents off the beat was strongly dependent on the absolute number of missing beats (for example, 5 accents off the beat could only occur when 3 beats were missing; see Table 1). To reduce problems with the inherent relation between missing beats and accents off the beat, we recoded the number of accents off the beat into three categories: few accents off the beat, some accents off the beat and many accents off the beat (see Table 1). *Type* of accents was either temporal or intensity. Finally, the number of years of *musical training* was included in the model as a continuous variable. All main effects and interactions between the four fixed factors were included in the model. Finally, to account for between-subject variation, we included a normally distributed random intercept for each participant.

All rhythms with no missing beats by definition also had no accents off the beat (see Table 1), making it impossible to use a full factorial design. To take this into account in the analysis, we included both polynomial and simple contrasts for the factor ‘beats missing’ in the regression. The condition with 0 beats missing was compared to the other three conditions combined using a simple contrast, to look at the difference between strictly metric rhythms (i.e., without any counterevidence) and rhythms with some degree of syncopation. To examine the effect of different amounts of counterevidence, the conditions with 1, 2 or 3 beats missing were compared using polynomial contrasts. Polynomial contrasts were also used for accents off the beat. The statistical analysis was conducted using R [60], and the clmm() function of the *ordinal* package [61] was used.

In addition to likelihood ratios (*χ*^2^) and *p*-values, below we also report *η*^2^ as a measure of effect size. Mixed models enjoy no universally-accepted standardized measurements of effect size (see [62] among others), and the challenges involved are even greater for ordinal models, which must assume that the underlying latent variable has some constant degree of dispersion and thus must rescale themselves for every added predictor. In the absence of a better method, here we approximate *η*^2^ from likelihood-ratio tests using Friedman’s [63] traditional formula for *χ*^2^ statistics:

$$\sqrt{\chi^{2}/N}$$

for one degree of freedom and

$$\sqrt{\frac{\chi^{2}}{\chi^{2}+N}}$$

for more than one degree of freedom. Although this is far from a perfect solution, with such a large sample, we feel that these approximate *η*^2^ values are still better than examining *p*-values alone.

### Results

**Fig 3:**
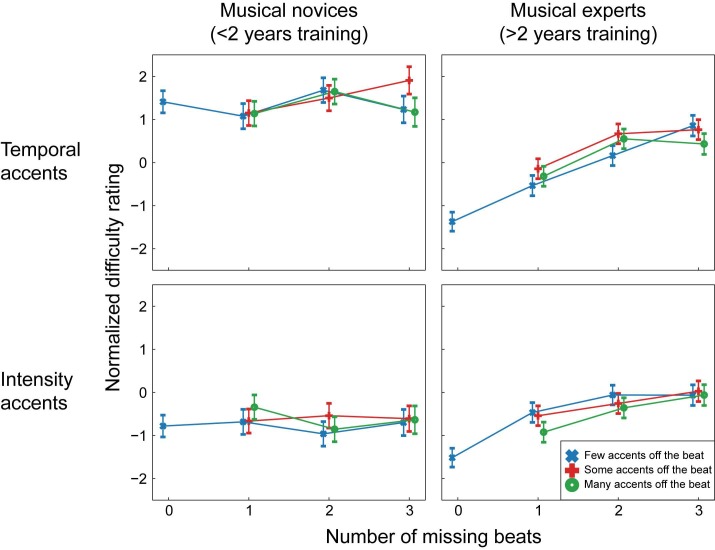
Estimated normalized ratings for all conditions in Experiment 1. For ease of visualization, participants were divided into two groups for this figure. Estimates are given for participants with less than 2 years of musical training (musical novices) and participants with more than 2 years of musical training (musical experts). Note that in the statistical analysis, musical training was included as a continuous variable and participants were not divided into groups. In the normalized scale, negative numbers indicate “easier to tap to” and positive numbers indicate “more difficult to tap to”. Error bars indicate 2 standard errors.

Fig 3 depicts the estimated normalized difficulty ratings for each condition. For ease of visualization, in Fig 3 participants were divided into 2 groups of musical training levels, but note that all statistical analyses were done with musical training as a continuous variable. In the figure, estimates are given separately for participants with less than 2 years of musical training and participants with more than 2 years of musical training to show the difference between those that can be considered real musical novices (a maximum of one year of music lessons) and those that have had more extended training (a minimum of three years of music lessons; no participants reported exactly two years of music lessons). This split in participants was not used for the analysis, but only for visualization purposes. Table 2 contains the results of the ordinal regression.

**Table 2:** Results of the ordinal regression in Experiment 1 and Experiment 2.

	Experiment 1				Experiment 2			
	LR (*χ*^2^)	df	*η*^2^	p	LR (*χ*^2^)	df	*η*^2^	p
**Missing beats**	256.87	3	0.046	<0.001***^#^	236.75	3	0.039	<0.001***^#^
**Accents off the beat**	6.09	2	0.001	0.05*	5.58	2	<0.001	0.06
**Type**	314.66	1	0.059	<0.001***^#^	64.22	1	0.011	<0.001***^#^
**Musical training**	5.55	1	0.001	0.02*	0.17	1	<0.001	0.68
**Missing beats** * **Accents off the beat**	2.50	4	<0.001	0.64	0.43	2	<0.001	0.81
**Missing beats** * **Type**	16.01	3	0.003	0.001**^#^	10.52	3	0.002	0.01*^#^
**Accents off the beat** * **Type**	4.25	2	<0.001	0.12	5.46	2	<0.001	0.07
**Missing beats** * **Musical training**	81.33	3	0.015	<0.001***^#^	55.73	3	0.010	<0.001***^#^
**Accents off the beat** * **Musical training**	0.01	2	<0.001	0.99	0.84	2	<0.001	0.66
**Type** * **Musical training**	57.30	1	0.011	<0.001***	1.99	1	<0.001	0.16
**Missing beats** * **Accents off the beat** * **Type**	8.26	4	0.002	0.08	0.24	2	<0.001	0.89
**Missing beats** * **Accents off the beat** * **Musical training**	0.91	4	<0.001	0.92	7.10	2	0.001	0.03*
Missing beats * Type * Musical training	2.71	3	<0.001	0.44	3.39	3	<0.001	0.34
**Accents off the beat** * **Type** * **Musical training**	-0.22	2	<0.001	1.00	0.73	2	<0.001	0.69
**Missing beats** * **Accents off the beat** * **Type** * **Musical training**	1.19	4	<0.001	0.88	0.83	2	<0.001	0.66

The test statistic for the ordinal regression is the Likelihood Ratio (or *χ*^2^), which represents the likelihood of the data under a model that includes the effect compared to a model that does not include the effect.

*Significant at *p*<0.05

**Significant at *p*<0.01

***Significant at *p*<0.001.

^#^Significant in both Experiment 1 and Experiment 2.

LR = Likelihood Ratio. df = degrees of freedom.

In general, with increasing numbers of missing beats (e.g., with fewer accents on the beat), rhythms were rated as progressively more difficult, as apparent from the main effect of beats missing (see Table 2 for test results). In addition, intensity rhythms were rated as easier than temporal rhythms (main effect of type), and participants with more musical training rated rhythms as easier to tap to than participants with less musical training (main effect of musical training). However, these main effects were accompanied by significant interactions, indicating that the relationship between beats missing, type, musical training, and difficulty ratings was interdependent.

**Fig 4:**
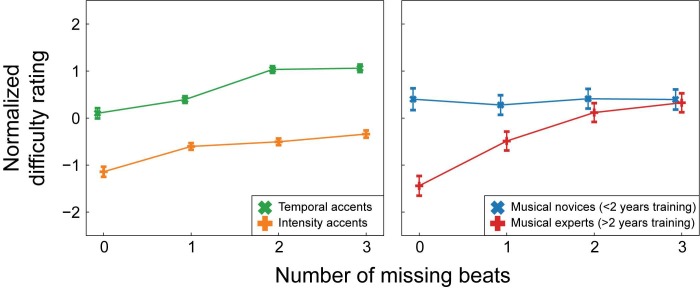
Interactions between beats missing and type and between beats missing and musical training in Experiment 1. As in Fig 3, for the chart on the right, depicting the interaction between musical training and beats missing, participants were divided into two groups for ease of visualization. Estimates are given for participants with less than 2 years of musical training (musical novices) and participants with more than 2 years of musical training (musical experts). In the statistical analysis, musical training was included as a continuous variable and participants were not divided into groups. For both charts, note that in the normalized scale, negative numbers indicate “easier to tap to” and positive numbers indicate “more difficult to tap to”. Error bars indicate 2 standard errors.

The regression yielded a very small but significant interaction between missing beats and type (see Table 2 and Fig 4). Planned contrasts showed that the linear association between the number of missing beats and the normalized difficulty was larger for temporal than for intensity rhythms (*z* = 2.44, *p* = 0.01, *r* = 0.03). In addition, there was a larger negative quadratic association between number of missing beats and normalized difficulty in temporal than intensity rhythms, showing that for temporal rhythms the increase in difficulty associated with more missing beats showed some curvature, and was larger from 1 to 2 missing beats than from 2 to 3 missing beats (*z* = 2.51, *p* = 0.01, *r* = 0.03). For the simple contrast, comparing the difficulty of the rhythms with 0 beats missing and rhythms with 1 or more beats missing, the interaction with type was not significant.

The interaction between missing beats and type shows that participants were more sensitive to the number of accents on the beat in temporal than intensity rhythms. More specifically, participants differentiated between rhythms with between one and three beats missing to a greater degree in temporal than intensity rhythms. Participants differentiated equally well between rhythms with no beats missing at all (e.g., strictly metric rhythms) and rhythms with one or more beats missing in both temporal and intensity rhythms.

A small but significant interaction was also found between missing beats and musical training (see Table 2 and Fig 4). The linear association between the number of missing beats and the normalized difficulty became larger with more years of musical training (*z* = 4.97, *p*<0.001, *r* = 0.07). The difference between normalized difficulty for rhythms with and without missing beats (the simple contrast) also became larger with more years of musical training (*z* = 7.51, *p*<0.001, *r* = 0.10). In general, participants rated rhythms as easier to perceive a beat in when fewer beats were missing and more beats were marked by an accent. The interaction between missing beats and musical training shows that musical training enhanced this effect.

A third interaction was found between type and musical training (see Table 2). Participants with little musical training rated the intensity rhythms as easier than the temporal rhythms, regardless of the presence of missing beats and accents off the beat. The difference in ratings between intensity and temporal rhythms became smaller with more years of musical training (*z* = 6.65, *p*<0.001, *r* = 0.09), showing that participants with many years of musical training rated the intensity and temporal rhythms as equally hard. Finally, a main effect was found for accents off the beat (see Table 2). However, none of the planned contrasts for this factor were significant, and the effect size for the main effect was extremely small.

## Experiment 2

We controlled the rhythms in Experiment 1 for the number of events and accents, and we allowed a maximum of five consecutive events. However, because of the constraints we used, all temporal rhythms with no beats missing in fact had a maximum of three consecutive events, while in the other conditions, some rhythms could contain four consecutive events (see Fig 1). Thus, rhythms in different conditions differed slightly in the distribution of events, creating some rhythms that had a higher local event density. Event density in rhythm has been associated with beat salience and the urge to move to a rhythm [64] and may have thus influenced our ratings. Moreover, all temporal rhythms with no beats missing consisted of five sixteenth notes, two eighth notes, one dotted eighth note and one quarter note, while the distribution of intervals in the other rhythms was more varied. In Experiment 2, we aimed to validate the results from Experiment 1 using the same procedure, while controlling for the possible effects of event density by only including rhythms that had a maximum of three consecutive events. Differences in interval distribution were also controlled, by allowing only rhythms with the same interval distribution that occurred in the strictly metric rhythms (e.g., rhythms without any counterevidence).

### Methods

#### Participants

We retrieved the data for Experiment 2 from the online application on February 6, 2015. At that time, 217 people had viewed the start page of the online application for Experiment 2, of whom 84 people had proceeded by providing consent and 67 had filled in their age and years of musical training. Among these, 53 people had read the instructions, 51 had listened to the examples, and 48 had rated one or more rhythms in the online application. There were 25 participants who had rated 60 or more rhythms and were thus included in the analysis, a 48-percent dropout rate. The remaining participants were on average 30.8 years old (range 20–69 years, SD = 11.8) and on average had had 7.0 years of musical training (range 0–25 years, SD = 6.4). The study was approved by the Ethics Committee of the Faculty of Humanities of the University of Amsterdam and the Non-Medical Research Ethics Board of the University of Western Ontario.

#### Stimuli

The stimuli were generated in exactly the same way as for Experiment 1, but with two extra constraints on the temporal rhythms: Only rhythms with no more than three consecutive events and only rhythms consisting of five sixteenth notes, two eighth notes, one dotted eighth note and one quarter note were included. With the extra constraints, some combinations of counterevidence in the temporal rhythms became impossible. The conditions with the combination of many accents off the beat and either one or three beats missing were thus excluded in Experiment 2. Table 3 shows the total possible rhythms within the constraints of Experiment 2 and the number of concatenated rhythms randomly constructed to use in the experiment. Note that all rhythms that were used in Experiment 2 could also have occurred in Experiment 1, but not all rhythms that were generated in Experiment 1 were allowed in Experiment 2. From the 296 randomly chosen rhythms in Experiment 2, 57 rhythms also occurred in Experiment 1 (19 percent).

**Table 3:** Characteristics of the rhythms used in Experiment 2.

Missing beats	Accents off the beat	Possible 16 grid-point rhythms		Number of concatenated 32 grid-point rhythms used in Experiment 2		Description of number of accents off the beat	No
		Temporal	Intensity	Temporal	Intensity		
**0**	0	12	6	28	28	Few	1
**1**	0	16	6	24	24	Few	2
	1	10	18	24	24	Some	3
	2	0	6	0	0	Many	
**2**	0	10	3	0	0	Not used	
	1	14	12	18	18	Few	5
	2	14	22	18	18	Some	6
	3	7	16	18	18	Many	7
	4	0	3	0	0	Not used	
**3**	2	4	2	0	0	Not used	
	3	7	8	10	10	Few	8
	4	9	12	8	8	Some	9
	5	0	6	0	0	Many	
**Total number of rhythms**				296			

In Experiment 2, two extra constraints were put on the temporal rhythms, to control for an uneven distribution of intervals and event density. Due to these extra constraints, we could not construct temporal rhythms with one beat missing and two accents off the beat (condition #4) and rhythms with three beats missing and five accents off the beat (condition #10). Therefore, these conditions were not used in Experiment 2. The numbers in the rightmost column correspond to the numbering for the conditions used in Fig 1.

#### Procedure and statistical analysis

The procedure and statistical analysis were identical to Experiment 1. In Experiment 2, a total of 6200 ratings were made. After excluding participants who rated less than 60 rhythms, 5771 ratings were included in the analysis. Fig 2A shows the distribution of the data for Experiment 2 and Fig 2B shows the normalization obtained with the ordinal regression.

### Results

**Fig 5:**
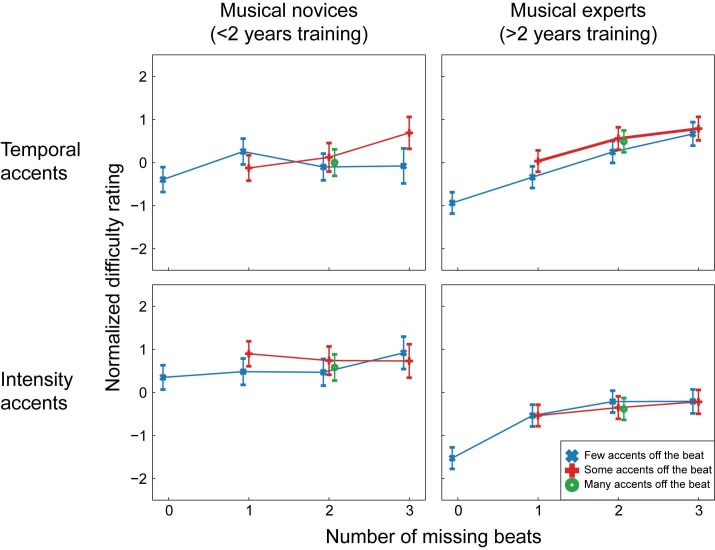
Estimated normalized ratings for all conditions in Experiment 2. As in Figs 3 and 4, for ease of visualization, participants were divided into two groups for this figure. Estimates are given for participants with less than 2 years of musical training (musical novices) and participants with more than 2 years of musical training (musical experts). Note that in the statistical analysis, musical training was included as a continuous variable and participants were not divided into groups. In the normalized scale, negative numbers indicate “easier to tap to” and positive numbers indicate “more difficult to tap to”. Error bars indicate 2 standard errors.

**Fig 6:**
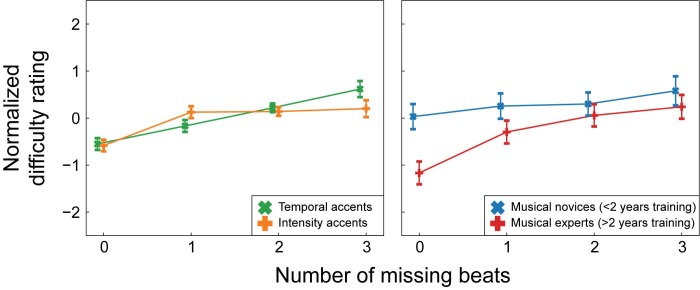
Interactions between beats missing and type and between beats missing and musical training in Experiment 2. As in Fig 4, for the chart on the right, depicting the interaction between musical training and beats missing, participants were divided into two groups for ease of visualization. Estimates are given for participants with less than 2 years of musical training (musical novices) and participants with more than 2 years of musical training (musical experts). Note that in the statistical analysis, musical training was included as a continuous variable and participants were not divided into groups. Note that for both charts, in the normalized scale, negative numbers indicate “easier to tap to” and positive numbers indicate “more difficult to tap to”. Error bars indicate 2 standard errors.

The estimated normalized difficulty ratings for each condition are shown in Fig 5 and the results of the ordinal regression can be found in Table 2. For visualization purposes only, the results are depicted separately for musical novices (<2 years of musical training) and musical experts (>2 years of musical training). In the statistical analysis, musical training was included as a continuous variable and participants were not divided into groups. We consider effects that replicate over both experiments, and thus appear reliable, as the most interesting. Therefore, Fig 6 depicts the interactions we found in both experiments.

Similar to Experiment 1, we found main effects of beats missing and type (see Table 2 for test results). The main effect of musical training did not reach significance. As in Experiment 1, these main effects were hard to interpret in the light of several significant interactions that were present.

A small but significant interaction was observed between missing beats and type (see Table 2 and Fig 6). The linear association between the number of missing beats and the normalized difficulty rating was larger for temporal than intensity rhythms (*z* = 2.63, *p* = 0.01, *r* = 0.04). As in Experiment 1, the interaction between the simple contrast and type was not significant. Thus, participants were more sensitive to the number of accents on the beat in temporal than intensity rhythms, but only when differentiating between rhythms with some degree of syncopation (e.g., with one or more beats missing). With more beats missing, rhythms were rated as more difficult to perceive a beat in and this effect was larger for temporal then intensity rhythms. Participants differentiated between rhythms with no beats missing (e.g., strictly metric) and rhythms with one or more beats missing (e.g., more or less syncopated) equally well in the temporal and intensity rhythms.

A three-way interaction was observed between missing beats, accents off the beat and musical training (see Table 2). The interaction between missing beats and accents off the beat increased with more years of musical training (z = 2.12, *p* = 0.03, *r* = 0.03). Musical novices rated rhythms with some accents off the beat as slightly more difficult than those with few accents off the beat regardless of the number of beats missing. However, musical experts rated rhythms with some accents off the beat as more difficult than those with few accents off the beat only when one beat was missing, but not when two or three beats were missing.

Although this three-way interaction was significant, its effect size was very small and this interaction was not found in Experiment 1. Thus, the practical use and reliability of this effect is questionable. Therefore, the much larger two-way interaction between missing beats and musical training (see Table 2 and Fig 6), which was found in Experiment 1 and replicated in Experiment 2, is of more interest. As in Experiment 1, in Experiment 2, musical experts differentiated more strongly between rhythms with and without missing beats than musical novices (z = 6.95, *p*<0.001, *r* = 0.09). Contrary to Experiment 1, in Experiment 2 the interaction between type and musical training did not reach significance.

Finally, to obtain some estimate of the validity of our experiments, we calculated how much variance our model explained for both experiments, correcting for the systematic differences between users (the random intercept in the models). In Experiment 1, the proportion of variance explained by our model was 0.17, while for Experiment 2, this was 0.08.

## Discussion

In this study we explored how different types of accents in musical rhythm influence the ease with which listeners with varying musical expertise infer a beat from a rhythm. Both in Experiment 1 and Experiment 2, musical training increased the sensitivity of participants to the number of accents on the beat. Participants rated rhythms with fewer missing beats (i.e., with more accents on the beat) as progressively easier to find a beat in. Musical training enhanced this effect. Contrary to our expectations, this greater sensitivity in musical experts was not selective to temporal rhythms, but also existed for intensity rhythms. Although musical training is not thought to be necessary for beat perception to develop [9,20], training does seem to affect how a listener processes the structure of accents that indicates where the beat is.

In many previous studies using stimuli designed after [30], the effect of musical training on the detection of a beat was not reported [18,30,65] or only musicians were tested [19]. Grahn and Brett [17] did examine the effect of musical training on the detection of a beat in temporal rhythms and did not find significant differences between musicians and non-musicians. However, they used a discrimination task, which implicitly probed beat perception. In a similar study, in which participants rated beat presence, differences were found between musicians and non-musicians [42]. That rating task strongly resembled the current task, as it required an explicit rating. Thus, musical novices may be capable of detecting a beat just as well as musical experts but may have less explicit access to the information required to make a rating of beat presence. In line with this, other work has shown that musical training enhances beat perception only when people attend to rhythm, but not when they ignore it [21]. As such, some aspects of beat perception may be more automatic, and independent of musical training, while aspects of beat perception that are related to attention and awareness may be enhanced by training. Future studies could examine potential differences between beat perception and beat awareness in musical novices and experts.

The current experiments suggest that musical experts are more able to use the regular structure of accents to infer a beat. This is in line with the finding that musical experts are more able than musical novices to use not only negative evidence, but also positive evidence, to infer metrical structure [55]. Musically trained listeners likely also have had more exposure to music than musical novices. They may therefore have stronger a priori expectations for duple metrical structures, as were used in the current experiments. Thus, the (mis)match between accents in the rhythm and the perceived metrical structure may have been larger for musically trained than untrained participants.

In both Experiment 1 and 2, participants were more sensitive to the number of accents on the beat in temporal than in intensity rhythms. The effect size of this interaction was extremely small, which warrants some caution in interpreting its practical use. Nonetheless, the interaction was highly significant in both experiments, with independent participants, and as such, seems reliable. The greater the number of beats missing in a rhythm, the more difficulty participants reported in finding a beat. This effect was larger for temporal than intensity rhythms. However, the results from the planned contrasts suggest that the effect of missing beats on the ratings was not just quantitatively but also qualitatively different for temporal and intensity rhythms. While the interaction between beats missing and type was significant for rhythms with one or more beats missing (as tested with the polynomial contrast), participants differentiated between rhythms with no beats missing and rhythms with one or more beats missing (as tested with the simple contrast) equally well for both types of rhythms.

Thus, listeners did differentiate between intensity rhythms that were strictly metric (e.g., did not contain any counterevidence) and intensity rhythms that contained some syncopation (e.g., some counterevidence), but they did not seem to differentiate between different degrees of syncopation in the intensity rhythms to the same extent as in the temporal rhythms. This may indicate that the Povel and Essens model [30] cannot be translated completely to rhythms with intensity accents. As these types of accents are commonly used in real music, studies of beat perception with only temporal rhythms may not provide a full picture of the mechanisms of beat perception in music. Grahn and Rowe [42] found that the brain networks involved in beat perception differed between intensity rhythms and temporal rhythms, and in the current study responses to the two types of rhythms were qualitatively different. More research is needed to understand how a beat is induced by music, where acoustic information as well as temporal cues are important.

In Experiment 1, musical novices, as expected, rated temporal rhythms as more difficult than intensity rhythms. This effect was generalized over all rhythms and was not modified by the amount of counterevidence. Musicians are more sensitive to the grouping rules that indicate temporal accents than non-musicians [66]. Thus, those with little musical training may have found it more difficult to extract information from the temporal rhythms than those with more musical training. In addition, musical novices attend more to lower (faster) levels of regularity in a metrical structure than musical experts [48,49]. In the intensity rhythms, all subdivisions of the beat contained a sound, creating an explicit isochronous pattern at a faster rate than the beat. Participants with little musical training may have focused on this lower level of regularity in judging how easy it was to hear a beat and may have ignored the accents altogether at the hierarchically higher level of the beat, whereas participants with more extended musical training may have been more attuned to events at all levels of the metrical hierarchy. The interaction between type and musical training, however, was absent in Experiment 2, and must thus be interpreted with caution. In the more restricted set of rhythms used in Experiment 2, the variability in the temporal rhythms was less than in Experiment 1, as we controlled for event density and the distribution of the temporal intervals used. The temporal rhythms in Experiment 2 were therefore more similar to each other than in Experiment 1, and this may have allowed participants to learn to recognize the intervals that were used. This may have made it generally easier for the musical novices to understand the grouping structure of the rhythm and may have therefore eliminated the difference between the two types of rhythms. It must also be noted that the sample of participants may have differed between the two experiments in ways that we cannot know. Generally, participants in Experiment 2 reported fewer years of musical training than in Experiment 1. However, it may be that they had superior beat perception abilities because of more exposure to musical rhythm [53] or innate ability [46].

The effects of accents off the beat were not consistent over the two experiments, with a main effect in Experiment 1 and an interaction between accents off the beat, missing beats and musical training in Experiment 2. In both experiments, the effect sizes for the influence of accents off the beat were extremely small. This is in line with Dynamic Attending Theory, which predicts more attentional resources on the beat and less detailed processing off the beat [33]. However, the weak results for counterevidence off the beat may also have been due to the design of the experiment. The difficulty ratings made by musical experts for temporal rhythms do show a numerical trend in the expected direction, with higher difficulty ratings for rhythms with more counterevidence off the beat. This effect weakens when rhythms become very complex (e.g., when 3 beats are missing). The effects of accents off the beat thus seem to be present only for musical experts, and only for rhythms with little counterevidence on the beat, hence the three-way interaction between accents off the beat, missing beats and musical training in Experiment 2. As the effect of accents off the beat thus is present only in a small subset of the total rhythms (only in 2 of the 8 conditions used in Experiment 2, and only for musically trained participants), the experiments may have lacked the power to detect the effects of counterevidence off the beat consistently.

The lack of an effect of accents off the beat in musical novices and for rhythms with many beats missing can be explained in two ways. First, it is possible that listeners do not differentiate between rhythms once it becomes too difficult to infer a beat. Thus, when three beats are missing, no beat is induced, and any further counterevidence created by accents off the beat cannot reduce beat induction any further. This ceiling effect may also explain the slight curvature in the effect of missing beats. While the difference between no counterevidence at all and some counterevidence is large, once it becomes harder to infer a beat, it does not matter whether more counterevidence is added.

A second explanation for the weak effects we found for accents off the beat may be that instead of perceiving a rhythm as more complex, people may shift the phase of the beat when too much counterevidence is present. While the Povel and Essens model [30] always regards rhythms as a whole entity, in reality, the perception of a beat unfolds over time [5]. In rhythms with a lot of counterevidence (i.e., many silent beats and many accents off the beat), some sections may have contained accents off the beat that were regularly spaced (see Fig 1). In such very complex rhythms it is possible that no beat was detected at all. By locally phase-shifting the perceived beat, or by changing the perceived period, a listener could find a new beat and make the rhythm appear less complex. This may have been easier for musical experts than musical novices. While the effects of accents off the beat were extremely small in our study, the possibility of local phase shifts may be worth considering in stimulus design. If only the number of missing beats is taken into account, beat perception in rhythms that are regarded as very complex (cf. [65]) may in fact be very easy when accents off the beat allow for phase shifting of the beat. A general and important challenge for future models of beat perception is to account for its inherently temporal nature to approximate human listening.

Two caveats in our stimulus design must be noted. First, the difference between temporal and intensity rhythms in our study can be characterized not only by the nature of the accents, but also by the presence of marked subdivisions in the rhythms. In the intensity rhythms all subdivisions of the beat contained a sound, while in the temporal rhythms some subdivisions were silent. When all subdivisions are marked, which is often the case in real music, people may rely less on accents indicating the beat and instead may infer a duple metrical structure from the isochronous subdivisions themselves (cf. [15,43]). This may explain why the effects of counterevidence in the current study were larger for temporal than intensity rhythms. One way of resolving this issue is by filling all silences in the temporal rhythms with sounds that are softer than the events that indicate the rhythmic pattern. Previously, Kung et al. [66] used such rhythms, but responses to these have not been compared to responses to temporal rhythms that do not contain all subdivisions. It is not clear whether the extraction of accents from temporal rhythms as proposed by [30] and used in the current experiment is the same as when all subdivisions are marked. This issue may be addressed in future research.

Second, we did not equate the different types of accents in terms of salience. However, it has been proposed that the subjective accents perceived in temporal patterns have an imagined magnitude of around 4 dB [4]. The physically present accents in the intensity rhythms were much larger (8.5 dB). Nonetheless, participants were more sensitive to the structure of the accents in the temporal rhythms than in the intensity rhythms. Thus, a discrepancy in salience between temporal and intensity accents would have led to an underestimation of this effect and is unlikely to have caused the effect.

For all the effects we report, effects sizes were small. In addition, the total amount of variance our model explains is also arguably small. This casts some doubt on the practical use of the model of how accents influence beat perception, and indicates that a large part beat perception is influenced by other factors. Some of these limitations may be inherent in the design of the study. For example, as has been noted before, the number of years of music lessons may not accurately estimate musical ability [67,68], which itself may also depend on exposure to different music styles [53], innate ability, and/or musicality [69]. Perhaps most importantly, as noted before, our model (like many) does not account for the fact that beat perception unfolds over time. Finally, as we used a web-based study, we did not have experimental control over possible strategies people may have used, and we cannot be certain that they adhered to the instruction not to move. These limitations suggest some caution in interpreting our results. However, it is reassuring that several findings were reliably replicated, even with an arguably simplified model of beat perception–most notably the interactions between accents on the beat and musical training, and accents on the beat and accent type. These effects may have a larger external validity as compared with effects found in lab-based experiments, precisely because we found them in both experiments despite all real-world variance and uncertainty caused by the Web-based setup [70,71].

## Conclusion

In the current study, we explored how the structure of different types of accents in rhythm influences the perception of a regular beat. Contrary to our expectations, both musical novices and musical experts were more sensitive to the structure of temporal accents than to the structure of intensity accents. As expected, musical training increased the sensitivity to the accent structure. Interestingly, beat finding in participants without musical training did not seem to be affected by the number of accents on the beat at all. The large effects of musical training on the perception of the beat may suggest that the use of stimuli with temporal accents in which the complexity is manipulated by varying the number of missing beats, as is often done, may not be meaningful to musical novices. The intensity accents as implemented in the current study did not improve beat perception for musical novices. However, a different combination of accents, and the use of statistical regularities in indicating the beat (see also [21]), may be more suited to their beat perception capacities. The use of non-temporal information in beat perception is not well understood and may be important to better understand this ability.

Our experiment provides a starting point in the use of online experiments to study beat perception. One could extend on this experiment by using a similar setup to obtain data from a larger group of people (for example through services like Amazon Turk). Ideally, this could result in a detailed model of how listeners with different backgrounds and experiences deal with different types of accents in rhythm as beat perception unfolds. Therefore, our experiment can be seen as the beginning of a search for stimulus material that is more ecologically valid, incorporates more musically relevant features, retains experimental control, and tests people varying in musical expertise and cultural background. Moreover, rhythms with a regular beat have been used in various clinical applications. Better understanding of what is needed for different populations to be able to extract a beat from a rhythm will help in designing more targeted and effective rehabilitation strategies using musical rhythm.

## Supporting information

S1 Sound Temporal rhythm with 0 beats missing and 0 accents off the beat. (MP3)

S2 Sound Temporal rhythm with 1 beat missing and 0 accents off the beat. (MP3)

S3 Sound Temporal rhythm with 1 beat missing and 1 accent off the beat. (MP3)

S4 Sound Temporal rhythm with 1 beat missing and 2 accents off the beat. (MP3)

S5 Sound Temporal rhythm with 2 beats missing and 1 accent off the beat. (MP3)

S6 Sound Temporal rhythm with 2 beats missing and 2 accents off the beat. (MP3)

S7 Sound Temporal rhythm with 2 beats missing and 3 accents off the beat. (MP3)

S8 Sound Temporal rhythm with 3 beats missing and 3 accents off the beat. (MP3)

S9 Sound Temporal rhythm with 3 beats missing and 4 accents off the beat. (MP3)

S10 Sound Temporal rhythm with 3 beats missing and 5 accents off the beat. (MP3)

S11 Sound Intensity rhythm with 0 beats missing and 0 accents off the beat. (MP3)

S12 Sound Intensity rhythm with 1 beat missing and 0 accents off the beat. (MP3)

S13 Sound Intensity rhythm with 1 beat missing and 1 accent off the beat. (MP3)

S14 Sound Intensity rhythm with 1 beat missing and 2 accents off the beat. (MP3)

S15 Sound Intensity rhythm with 2 beats missing and 1 accent off the beat. (MP3)

S16 Sound Intensity rhythm with 2 beats missing and 2 accents off the beat. (MP3)

S17 Sound Intensity rhythm with 2 beats missing and 3 accents off the beat. (MP3)

S18 Sound Intensity rhythm with 3 beats missing and 3 accents off the beat. (MP3)

S19 Sound Intensity rhythm with 3 beats missing and 4 accents off the beat. (MP3)

S20 Sound Intensity rhythm with 3 beats missing and 5 accents off the beat. (MP3)

S1 Fig Example of the interface used during the online experiment. (PDF)

S1 Data Dataset from both Experiment 1 and Experiment 2. Labels and descriptions can be found in the “readme” tab.
(XLSX)
