# Supplementary material for: What makes a rhythm complex? The influence of musical training and accent type on beat perception
Source: PLoS One. 2018 Jan 10;13(1):e0190322. doi: 10.1371/journal.pone.0190322 (PMC5761885; doi:10.1371/journal.pone.0190322)
Supplement: S1 Fig — (PDF) [file pone.0190322.s021.pdf]

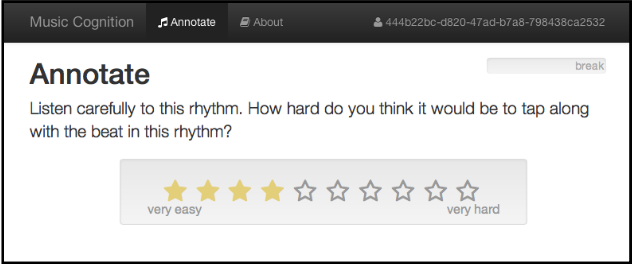
Music Cognition
Annotate
About
444b22bc-d820-47ad-b7a8-798438ca2532
break
Annotate
Listen carefully to this rhythm. How hard do you think it would be to tap along with the beat in this rhythm?
very easy
very hard
